# Pomegranate Seeds Extract Possesses a Protective Effect against Tramadol-Induced Testicular Toxicity in Experimental Rats

**DOI:** 10.1155/2020/2732958

**Published:** 2020-03-09

**Authors:** Fatma M. Minisy, Hossam H. Shawki, Abdelfatteh El Omri, Ahmed A. Massoud, Enayat A. Omara, Fatma G. Metwally, Manal A. Badawy, Neveen A. Hassan, Nabila S. Hassan, Hisashi Oishi

**Affiliations:** ^1^Department of Comparative and Experimental Medicine, Nagoya City University Graduate School of Medical Sciences, Nagoya, Japan; ^2^Department of Pathology, Medical Division, National Research Center, Cairo, Egypt; ^3^National Gene Bank of Egypt (NGB), Agricultural Research Center (ARC), Giza, Egypt; ^4^Department of Biological Sciences, Faculty of Science, King Abdulaziz University (KAU), Jeddah, Saudi Arabia; ^5^Department of Zoology, Faculty of Science, Tanta University, Tanta, Egypt; ^6^Department of Academic Science, Research Institute of Ophthalmology, Giza, Egypt

## Abstract

Tramadol is a centrally acting opioid analgesic that is extensively used. The chronic exposure to tramadol induces oxidative stress and toxicity especially for patients consuming it several times a day. Previously, we and others reported that tramadol induces testicular damage in rats. This study was conducted to investigate the possible protective effect of pomegranate seed extract (PgSE) against tramadol-induced testicular damage in adult and adolescent rats. Male rats were orally treated with tramadol or in a combination with PgSE for three weeks. Testes were then dissected and analyzed. Histological and ultrastructural examinations indicated that tramadol induced many structural changes in the testes of adult and adolescent rats including hemorrhage of blood vessels, intercellular spaces, interstitial vacuoles, exfoliation of germ cells in lumen, cell apoptosis, chromatin degeneration of elongated spermatids, and malformation of sperm axonemes. Interestingly, these abnormalities were not observed in tramadol/PgSE cotreated rats. The morphometric analysis revealed that tramadol disrupted collagen metabolism by elevating testicular levels of collagen fibers but that was protected in tramadol/PgSE cotreatment at both ages. In addition, DNA ploidy revealed that S phase of the cell cycle was diminished when adult and adolescent rats were treated with tramadol. However, the S phase had a normal cell population in the cotreated adult rats, but adolescent rats had a lower population than controls. Furthermore, the phytochemistry of PgSE revealed a high content of total polyphenols and total flavonoids within this extract; besides, the DPPH free radical scavenging activity was high. In conclusion, this study indicated that PgSE has a prophylactic effect against tramadol-induced testicular damage in both adult and adolescent ages, although the tramadol toxicity was higher in adolescent age to be completely protected. This prophylactic effect might be due to the high antioxidant compounds within the pomegranate seeds.

## 1. Introduction

There has been expanding global concerns on the decline of male fertility rates and testicular cancer in the past 3–5 decades [1, 2]. Along with the conventional causes of male infertility [3], exposures to certain analgesic drugs such as opioid narcotics induce oxidative stress and adversely affect male fertility [4–8]. Opioid analgesic drugs are known to be the most effective medication for moderate to serious pain for adult and young patients [9].

Tramadol hydrochloride (tramadol HCl) is one of the centrally acting opioid drugs that is extensively used parenterally and orally as a pain reliever [10]. It is rapidly absorbed and distributed throughout the body with a higher analgesic effect than the parent drugs [11]. Tramadol contributes to analgesic activity by working as agonists of the *μ*-opioid receptor and also by inhibiting serotonin and norepinephrine reuptake and therefore enhancing inhibitory effects on pain transmission in the spinal cord [12]. Tramadol is used to reduce pain resulted from posttraumatic, obstetric, osteoarthritis, fibromyalgia, renal, biliary colic, and neuropathic pain [12]. However, multiple tissue toxicities were reported when tramadol was administrated [13–17], especially for patients consuming tramadol medication several times daily to relieve chronic pain. We and others have shown that tramadol induces testicular toxicity in adults [5, 9, 18–20].

Tramadol like other opioids induces oxidative stress by decreasing the antioxidant levels in the body [21–23]. Oxidative stress ensues when the Reactive Oxygen Species (ROS) level and antioxidant defense system are imbalanced, which results in structural alterations of the cell and apoptosis [24, 25]. Recently, natural products have been used for the treatment of oxidative stress [26]. Several plants were suggested, such as pomegranate [27]. The pomegranate (*Punica granatum* L.) belongs to the family Punicaceae. This fruit has long been cultivated and widely consumed as fresh fruit or in beverage form. The edible parts of pomegranate fruit comprise 78% juice and 22% seed [28]. Pomegranate is rich in sugars, vitamins, polysaccharides, polyphenols, and minerals [29–31]. Pomegranate was used as a medicinal plant for long [27, 32, 33]. Its extract was found to increases the antioxidant capacity *in vivo* and *in vitro* [34, 35]. This activity may be related to the diverse phenolic compounds present in pomegranate, including punicalagin isomers, ellagic acid, anthocyanins (3-glucosides and 3,5-diglucosides of delphinidin, cyanidin, and pelargonidin), and different flavanols [35–38]. Moreover, pomegranate juice consumption increases significantly sperm quality, spermatogenic cell density, and testosterone level in male rats [39].

Although the incidence that both adult and young patients are treated with tramadol, the risk factors of age-dependent treatment are poorly studied. Thus, the present study aimed to determine the risk level of tramadol treatment on the testicular structure at adult and adolescent ages and to evaluate the possible protective effects of pomegranate seed extract upon coadministration with tramadol at both ages.

## 2. Materials and Methods

### 2.1. Animals

This study was carried out on adult (six weeks old; weight 96 ± 19 g) and adolescent (three weeks old; weight 82 ± 20 g) male Wistar rats (*Rattus norvegicus*). Rats were bought from animal colony NRC-Egypt and were housed in a quite nonstressful environment for an acclimation period of one week prior to the study to be stabilized in the new environment. They were allowed for free access to food and water during the experimental period. The maintained lab diet was as standard (protein: 160.4, fat: 36.3, fiber: 41 g/kg, and metabolizable energy: 12.08 MJ). All animal experiments were performed in a humane manner upon approval by the Animal Experiment Committee of National Research Center, Egypt, and Nagoya City University, Japan. Moreover, they were euthanized with carbon dioxide gas to minimize animal suffering.

### 2.2. Chemicals

Tramadol hydrochloride (tramadol HCl), 225 mg tablets, was obtained from Mina-Pharm, Egypt. The LD_50_ values of tramadol administration orally were estimated to be 300–350 mg/kg body weight for rats [40]. The treated doses used of this drug were calculated as previously reported [41], which were nearly comparable to the human effective therapeutic doses. Tramadol was dissolved in distilled water and given by stomach tube during the experimental period.

### 2.3. Preparation of Pomegranate Extract (PgSE)

Pomegranate seeds were collected, dried, crushed to fine powder, and mixed with 70% ethanol for 24 hours with repeated stirring. Ethanolic extract was obtained by repeating the extraction procedure for 3 successive times. The resulting ethanol extracts were subsequently filtered and concentrated with a vacuum rotary evaporator (Heidolph®.VV2000) under reduced pressure at a temperature of 55°C and then the residues were lyophilized using a vacuum freeze drier (Tilburg, Holland; 145Fm-RB). The lyophilized powder was diluted in distilled water on the day of treatment. The treated doses used for PgSE were as previously reported [42]. Each rat received 40 mg/kg body weight in a total volume of 1 ml by stomach tube during the experimental period.

### 2.4. Experimental Design

The experimental model of the current study was as shown in [Supplementary-material supplementary-material-1]. Thirty-six male rats were divided according to their age into two main groups: 18 adult rats and 18 adolescent rats. Each group was then subdivided into three groups (six animals per group). The control group received orally 1 ml of 0.9% saline. The treated group received orally tramadol dose of 20, 40, and 80 mg/kg during the 1^st^, 2^nd^, and 3^rd^ week, respectively. The cotreated group received orally 40 mg/kg PgSE in addition to the tramadol dose.

### 2.5. Tissue Collection and Histological Analysis

Animals were sacrificed and testis samples were incised and fixed immediately in 10% phosphate-buffered formalin (pH 7.4) for 48 h. Tissues were then processed routinely for paraffin embedding. Sections with 5 *μ*m thickness were cut and mounted on glass slides. After deparaffinization, slides were stained with hematoxylin and eosin (H&E), examined under bright field light microscopy, and photographed.

### 2.6. TUNEL Assay

Testicular apoptotic cells were examined by Terminal deoxynucleotidyl transferase dUTP Nick end labeling (TUNEL) assay using Invitrogen Click-iT Plus TUNEL imaging kit from Invitrogen (C10618) following the manufacturer's protocol. Briefly, testis sections of 5 *μ*m thickness were deparaffinized, fixed in 4% PFA for 15 min, and permeabilized with Proteinase K for another 15 min. The TdT reaction cocktail was added and incubated for 1 h, followed by 30 min incubation with the Click-iT reaction cocktail. The sections were then counterstained using DAPI and observed with an Olympus FV3000 confocal laser scanning microscope. For quantitative analysis, at least 6 sections of each testis from three rats within each group were examined.

### 2.7. Transmission Electron Microscopy (TEM)

Testis samples were cut into small blocks, fixed in 2.5% glutaraldehyde for 3 h at 4°C, and postfixed in 1% osmium tetroxide for one hour at room temperature. The tissues were dehydrated through a graded ethanol series and embedded in Epon 812. The blocks were then cut into ultrathin sections with LKB ultramicrotome and stained with uranyl acetate followed by lead citrate. Sections were examined using a transmission electron microscope (Model JEM-1400 Plus). Flagella with axonemal disorganization were counted in 5 random fields of each group and presented as a percentage.

### 2.8. Morphometric Analysis of Collagen Fibers

Testis sections (5 *μ*m) were stained with Masson's trichrome for the quantification of collagen fibers according to Drury and Wallington [43]. The area percentage of collagen fibers was measured in four sections of each testis from three rats within each group. The percentage of positive reaction was measured in ten random fields of each section (FOV = 25 mm^2^) at high power (400x) using a computer-assisted image analyzer (Qwin Leica image processing analysis system, Cambridge, England). Measurements were made based on the intensity of blue color which represents the collagen density.

### 2.9. DNA Ploidy Analysis

Testis sections (5 *μ*m) were stained with Feulgen staining for DNA ploidy analysis. The nuclear integrated optical density (OD) which is the cytometric equivalent of DNA content was measured in four sections of each testis from three rats within each group. The intensity of the stain was measured in ten random fields of each section (FOV = 25 mm^2^) at high power (400x), using a computer-assisted image analyzer (Qwin Leica image processing analysis system, Cambridge-England). The DNA histograms were classified as previously described based on the amount of DNA relative to normal control [44].

### 2.10. Phytochemical Analysis of Pomegranate Extract

Pomegranate seeds extract was examined for its total phenolic content, total flavonoids content, and free radical DPPH scavenging activity. The total phenolic content was determined using the Folin-Ciocalteu colorimetric method [45]. Briefly, the extracts were oxidized with Folin-Ciocalteau reagent and then neutralized with aqueous Na_2_CO_3_ solution. After 40 min in dark, the absorbance was measured at 725 nm. The total phenolic content was determined using a calibration curve prepared and expressed as micrograms of gallic acid equivalents per gram of sample.

The total flavonoid content was quantified as previous [46]. Briefly, the extracts were mixed with sodium nitrite followed by adding aluminum chloride and sodium hydroxide. The optical absorbance was then measured at 510 nm. Total flavonoid was expressed as micrograms of gallic acid equivalents (GAEs) per gram of sample.

The antioxidant activity was determined by DPPH free radical scavenging assay as previous [47]. Briefly, the extracts were mixed with DPPH solution and incubated in the dark at room temperature for 1 h. The absorbance at 517 nm was measured against a blank of pure methanol. The antioxidant activity was determined by means of a calibration curve prepared with Trolox and expressed as micrograms of Trolox equivalent (TE) per unit weight of the sample. Percent of the DPPH free radical effect was calculated by the following equation:

(1) $$\begin{array}{c} \text{DPPH scavenging effect}\%=100\times \frac{\left(A_{\text{control}}-A_{\text{sample}}\right)}{A_{\text{control}}}. \end{array}$$

### 2.11. Statistical Analysis

The data obtained were compiled and statistically analyzed and expressed as mean ± standard deviation. The data normal distributions were examined by the Shapiro-Wilk test. Differences between groups were compared by ANOVA using the SPSS software (version 16). Tukey post hoc multiple comparison tests of significant differences among groups were determined. The probability value *p* < 0.05 was considered statistically significant.

## 3. Results

### 3.1. Histopathological Examination of Testes

**Figure 1:**
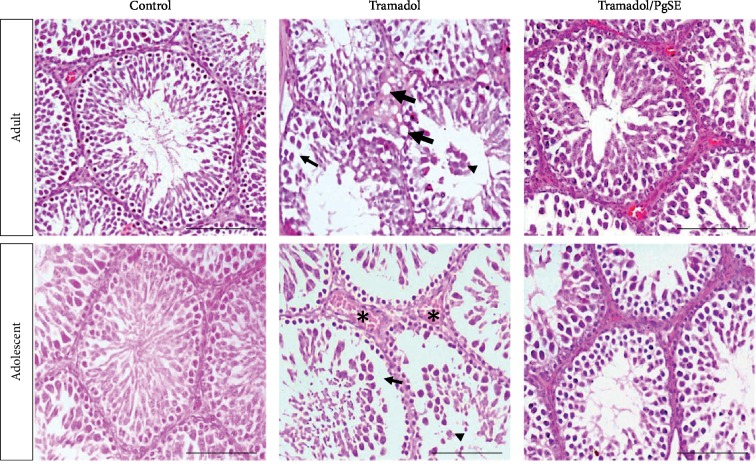
Histopathology of Tr-treated testicular tissues and cotreated with PgSE. Sections of adult and adolescent rat testes were examined via HE staining. Tr-treated group induced intercellular spaces within seminiferous (thin arrow), interstitial vacuoles (thick arrow), exfoliation of germ cells in lumen (arrowhead), and hemorrhage of blood vessels (star). Scale bars: 100 *μ*M.

The experimental model of the study was shown in [Supplementary-material supplementary-material-1]. Adult and adolescent male rats were treated orally with normal saline (saline-treated controls), tramadol (Tr-treated), or tramadol with pomegranate seed extract (Tr/PgSE cotreated) for 3 weeks period. The tramadol concentration was gradually increased from 20 to 80 mg/kg to simulate the physical dependence of the drug similar to the chronic use in humans to reach the effective doses. Animals were then sacrificed and testes were collected, sectioned, and stained with HE for histological analysis. Results indicated that several testicular abnormalities were induced when tramadol alone was administrated to mice in both adult and adolescent ages (Figure 1). Testicular sections from Tr-treated groups showed hemorrhage of blood vessels, intercellular spaces within seminiferous tubules, interstitial vacuoles, and exfoliation of germ cells in the seminiferous lumen. However, these testicular abnormalities were observed in neither saline-treated controls, PgSE-treated controls, nor Tr/PgSE cotreated groups at both adult and adolescent ages (Figure 1 and [Supplementary-material supplementary-material-1]).

### 3.2. Apoptosis of Testicular Cells

**Figure 2:**
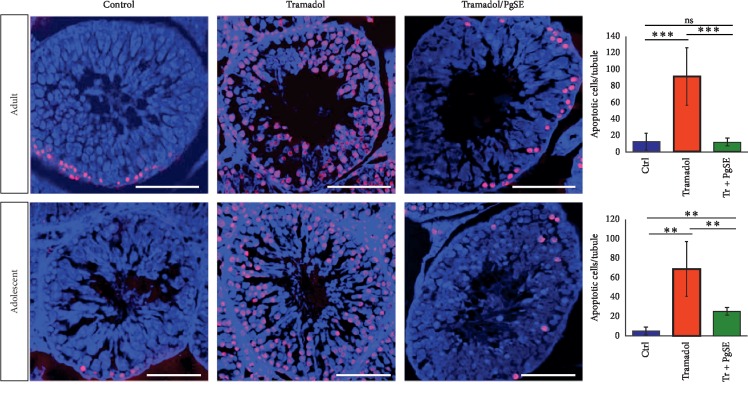
Testicular apoptotic cells of Tr-treated testicular tissues and cotreated with PgSE. Adult and adolescent rat testes were examined via TUNEL assay. TUNEL-positive cells are stained in red. The nuclei were counterstained with DAPI. The quantitative numbers of apoptotic cells were shown on the right. The data represent the mean ± SD. ^*∗∗*^*P* < 0.01 and ^*∗∗∗*^*P* < 0.001 mean statistical difference. Scale bars: 100 *μ*M.

To investigate whether the testicular abnormalities observed by HE were accompanied by apoptosis of the testicular cells, we carried out a Terminal deoxynucleotidyl transferase dUTP Nick end labeling (TUNEL) assay. TUNEL-positive cells were identified and enumerated from histological sections per seminiferous tubule (Figure 2). The TUNEL-positive cells were observed in saline-treated controls, and the numbers of these cells were significantly increased (*P* < 0.001 and *P* < 0.01) in Tr-treated groups of both adult and adolescent rats, respectively. Significant fewer apoptotic cells were detected in the Tr/PgSE cotreated adults (*P* < 0.001) and Tr/PgSE cotreated adolescents (*P* < 0.01) when compared to Tr-treated groups. The Tr/PgSE cotreated adults showed a nonsignificant difference with saline-treated controls. However, the number of apoptotic cells in Tr/PgSE cotreated adolescents was still statistically different from saline-treated controls (*P* < 0.01), although it was visibly lower than the Tr-treated group. The overall incidence of these results indicated that PgSE could protect against the cell death reprogramming by tramadol administration.

### 3.3. Ultrastructure Analysis of Testicular Cells

**Figure 3:**
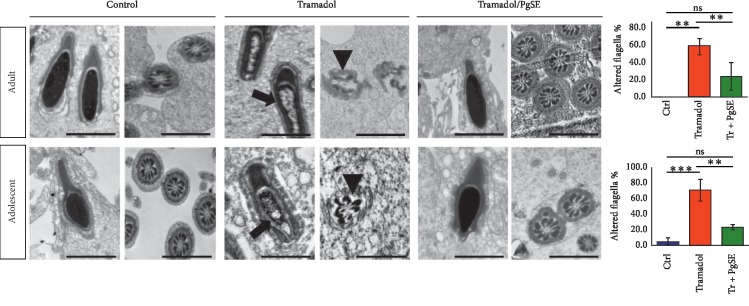
Ultrastructural analyses of Tr-treated testicular tissues and cotreated with PgSE. Adult and adolescent rat testes were examined via transmission electron microscopy. The Tr-treated group showed degeneration of elongated spermatid's chromatin (arrow) and malformation of sperm axonemal structure (arrowhead) in both adult and adolescent rats. In the cotreated groups, the abnormal ultrastructure appearances were not detected. The quantitative numbers of altered flagella were shown on the right. The data represent the mean ± SEM. ^*∗∗*^*P* < 0.01 and ^*∗∗∗*^*P* < 0.001 mean statistical difference. Scale bars: 1 *μ*M.

To investigate in-depth the effect of the tramadol and the Tr/PgSE cotreatment on haploid germ cells, the ultrastructure of the testicular tissues was characterized. Testis samples were cut into small blocks, fixed, embedded, ultrathin sectioned, and finally examined using a transmission electron microscope (Figure 3). Results indicated that Tr-treated groups of both adult and adolescent rats had a degeneration in the elongated spermatid's chromatin which appears unraveled in an empty perinuclear area due to shrinkage of the nuclear content comparing to saline-treated controls. In the Tr/PgSE cotreated groups, the elongated spermatids showed a pyriform nucleus with condensed chromatin and completely formed acrosome. The second type of defect we observed is a malformation of sperm axonemal structure in both adult and adolescent rats comparing to saline-treated controls. Cross section of spermatozoa from Tr-treated groups shows complete disruption and disorientation of axonemal structure microfilaments, mitochondrial membrane degeneration, and rapture of plasma membrane sheath. No abnormal ultrastructure phenomena were detected in Tr/PgSE cotreated groups at both ages. This suggests that the chromatin alteration in elongated spermatids and sperm axonemal malformation was related to tramadol toxicity and that could be protected by PgSE cotreatment.

### 3.4. Morphometric of Collagen Fibers in Testes

**Figure 4:**
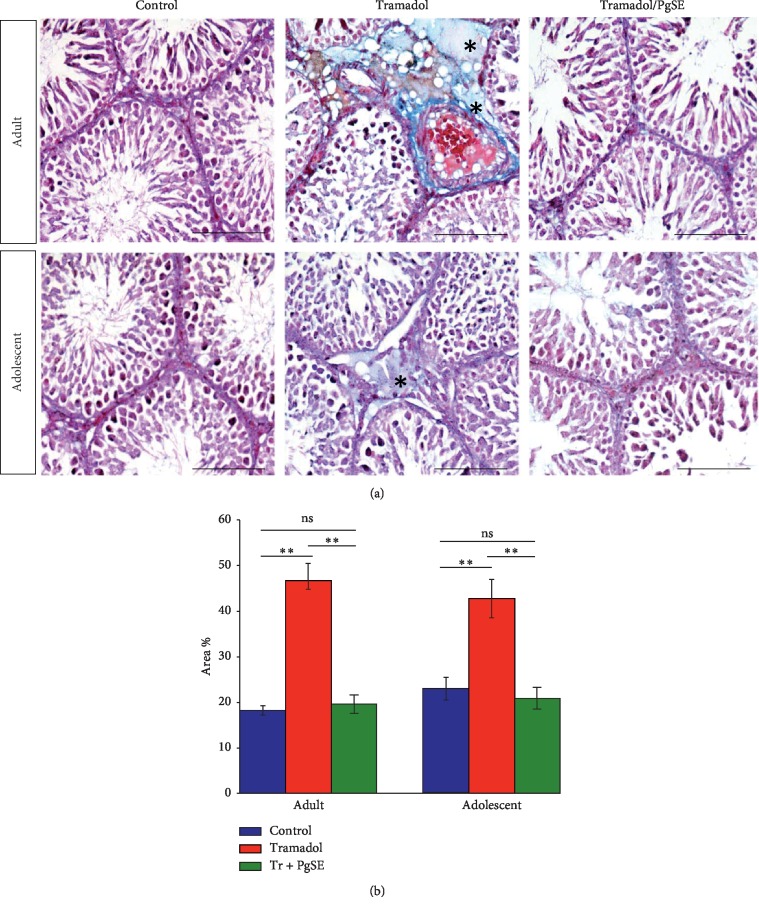
Morphometrical analyses of Tr-treated testicular tissues and cotreated with PgSE. (a) Adult and adolescent rat testes were examined via Masson's trichrome staining. The blue color represents collagen density (star). The Tr-treated group showed a significant increase in collagen fibers content in both adult and adolescent rats but not in the cotreated groups. Scale bars: 100 *μ*M. (b) The area percentage of collagen fibers measured in rat testes from each group was shown as bars. Data represent the mean ± SD, ^*∗∗*^*P* < 0.01. ns: nonsignificant.

The morphometrical analyses of the collagen fibers were examined by staining the testis sections with Masson's trichrome staining. A high degree of collagen fibers deposition surrounding the seminiferous tubules was observed in the testes of Tr-treated groups (Figure 4(a)). Quantification of collagen fibers from adult rat testes revealed that the measured area percentage of saline-treated control, Tr-treated, and Tr/PgSE cotreated groups were 22.97 ± 2.45, 42.82 ± 4.25, and 20.88 ± 2.45, respectively (Figure 4(b)). Quantification of collagen fibers from adolescent rat testes revealed that the measured area percentage of saline-treated control, Tr-treated, and Tr/PgSE cotreated groups were 18.18 ± 1.14, 46.68 ± 3.76, and 19.59 ± 2.08, respectively (Figure 4(b)). Tr-treated groups were significantly higher in collagen fibers (*P* < 0.01) compared to saline-treated control or Tr/PgSE cotreated groups at both adult and adolescent ages. But no statistical difference in collagen fibers area percentage was observed between saline-treated control versus Tr/PgSE cotreated groups in both adult and adolescent rat testes. That unequal collagen distribution indicated a tramadol-based disrupted metabolism.

### 3.5. DNA Ploidy of Testicular Cells

**Figure 5:**
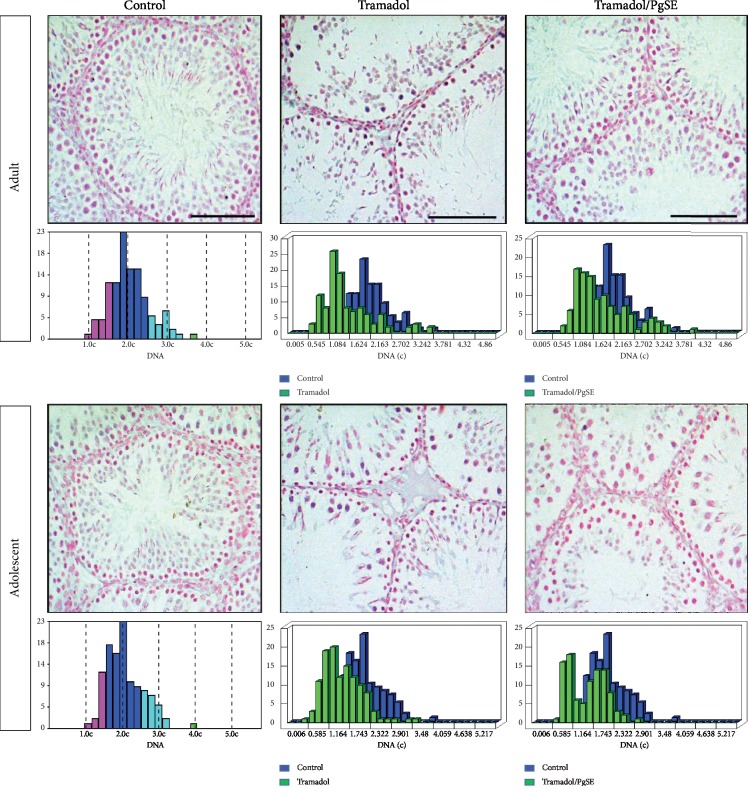
DNA ploidy image cytometry of Tr-treated testicular tissues and cotreated with PgSE. Adult and adolescent rat testes were examined via Feulgen staining. The nuclear integrated optical density (OD) which is the cytometric equivalent of DNA content was measured. The DNA histograms were classified based on the amount of DNA as haploid (<1.5c), diploid (1.5–2.5c), triploid (2.5–3.5c), and tetraploid (3.5–4.5c). The Tr-treated group had diminished the triploid cell population in both ages. Scale bars: 100 *μ*M.

The DNA ploidy analysis was examined by staining the testis sections with Feulgen staining (Figure 5). The DNA content per nucleus is calculated for all the nuclei from the measured sample to yield a histogram of the cell cycle distribution. The DNA histograms are classified into four cell populations: haploid (<1.5c), diploid (1.5–2.5c), triploid (2.5–3.5c), and tetraploid (3.5–4.5c) based on the amount of DNA. The results indicated that the triploid which represents the population at S phase was diminished in Tr-treated groups as compared with saline-treated control in both adult and adolescent ages. However, the Tr/PgSE cotreated group in adults showed similar S phase distribution to saline-treated control, but in the adolescent, the S phase population was lower than saline-treated control (Figure 5 and [Supplementary-material supplementary-material-1]). The overall results indicated that tramadol induces disruption of cell cycle progression and PgSE could protect against that effect in adult rats, but adolescent rats were more sensitive to be completely protected.

### 3.6. Phytochemical Analysis of PgSE

**Figure 6:**
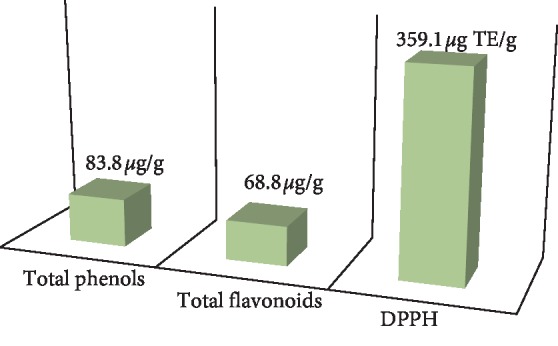
Phytochemistry of pomegranate seeds extract (PgSE). The PgSE was examined for its total phenolic content, total flavonoids content, and free radical DPPH scavenging activity. The phytochemical analyses indicated the presence of a high level of phenols, flavonoids, and DPPH. The measured values were written over the bars.

To understand the protective effect of PgSE against the tramadol toxicity, we analyzed the phytochemistry of PgSE which represents the antioxidant activities including total phenolic content, total flavonoids content, and free radical DPPH scavenging activity (Figure 6). The PgSE exhibited high total polyphenol content of 83.8 *μ*g/g (expressed as gallic acid equivalent) and high total flavonoid content of 68.8 *μ*g/g (expressed as gallic acid equivalent). The DPPH free radical scavenging of PgSE is 359.1 *μ*g TE/g and the percentage of DPPH inhibition effect was 210.4%.

## 4. Discussion

One of the main causes of male infertility is the exposure to opioid analgesic drugs that induce oxidative stress [4–9]. Tramadol is one of the opioid drugs that is extensively used as a pain-killer for chronic pain and cancers [10]. However, multiple tissue toxicities were reported for patients consuming tramadol medication several times a day [13–17]. Since tramadol like other opioids induces oxidative stress which alters cell structure causing apoptosis [21–25], the pomegranate was used in the current study as a protective agent based on its antioxidant activity [34, 35, 48, 49]. Thus, our study was focused on evaluating the toxicity of tramadol on testicular tissue from adult and adolescent ages and testing the protective effect of pomegranate seed extract (PgSE) against this toxicity.

Results revealed that tramadol had adverse effects on testes of both adult and adolescent rats with different ranges on the histopathology, ultrastructure architecture, morphometry, and DNA ploidy. Interestingly, the above-mentioned defects caused by tramadol were prevented when PgSE was coadministered with tramadol. The protective effect could be due to the bioactive compounds that are known to have multiple therapeutic effects such as tannins [50], anthocyanins [51], alkaloids [52], phenolic acids [53], estrogenic flavonoids [54], and conjugated fatty acids [55], which are found vigorously in pomegranate [56].

The histopathology of the adult and adolescent testes showed congested blood vessels, intercellular spaces within seminiferous tubules, interstitial vacuoles, and exfoliation of germ cells in lumen. These results were matched with previous studies [11]. It is known that testicular blood flow is controlled by testosterone, so any defect in Leydig cells alters the size of blood vessels [57]. Previous reports found that opiates including tramadol reduce the serum testosterone levels [58]. The congested blood vessels and interstitial vacuoles observed indicated testicular vascular atrophy and Leydig cell damage has occurred. The protective effect of PgSE shown in the cotreated groups indicated the ability of PgSE to prevent the damage of Leydig cells and restore testosterone to control levels as previously found with pomegranate juice [59]. Besides, the intercellular spaces within seminiferous tubules and exfoliation of germ cells indicated that germ cells may undergo apoptosis or they may lose the Sertoli-germ cell contact being shed into the seminiferous lumen and form intracellular spaces as previously described [60, 61]. In this connection, we examined the testicular cells apoptosis by TUNEL assay. Apoptosis has a critical role in the removal of damaged germ cells to prevent the formation of abnormal sperms. Results indicated that a significant increase of apoptotic cells was detected in testicular sections of both adult and adolescent rats when treated with tramadol. Moreover, a diffuse and defect in the chromatin condensation of elongated spermatids were found. This could be explained by the ability of tramadol to decrease the antioxidant levels in the testis which imbalance the antioxidant defense system leading to cell oxidative stress [9, 21, 23]. Oxidative damage involved inactivation of p53 which, in turn, mediates either DNA repair or apoptosis [62]. Tramadol was found to induce neurotoxic effects through alteration of p53, Bax, and Bcl-2 apoptotic pathway [63]. On the other hand, the cotreated groups have comparable apoptotic cell numbers to controls and normal spermatids' chromatin. Previous reports revealed that extracts from plants with antioxidant activity can reduce the apoptotic of germ cells caused by chemical toxicity [64, 65]. Similarly, pomegranate seed extract prevented germ cell apoptosis, possibly by modulating the ROS/Nrf2/p53 signaling cascade as previously described [66–68].

The ultrastructural examination showed complete degeneration and disorientation of axonemal structure microfilaments. This is predominantly due to the fact that sperms are rich in polyunsaturated fatty acids, rendering them highly susceptible to oxygen-induced damage and, hence, lipid peroxidation. Previous reports indicated that tramadol increases peroxidation of sperm lipids [9, 59], which in particular produces cytotoxic aldehydes [69]. These cytotoxic aldehydes were shown to inhibit a large number of cellular enzymes including glyceraldehyde-3-phosphate dehydrogenase [70], one of the key enzymes for the generation of ATP in mitochondria, and therefore a rapid loss of intracellular ATP occurs. The ATP depletion leads to damage of axonemal structure due to insufficient axonemal protein phosphorylation as previously reported [71, 72]. We found that the cotreatment of PgSE with tramadol could protect against the axonemal damage, and this could be due to the ability of pomegranate to inhibit testicular lipid peroxidation as previously revealed [59].

Additionally, a high degree of collagen fibers deposition in the extracellular matrix surrounding the seminiferous tubules was also observed in the adult and adolescent rat testes treated with tramadol indicating tramadol disrupted collagen metabolism. Nevertheless, the cotreatment of PgSE with tramadol has not revealed a high degree of collagen fibers deposition. Sertoli cells are involved in the deposition of extracellular matrix components such as collagen [73, 74]. Thus, the increased collagen fibers in Tr-treated groups might be a result of impaired Sertoli cell function or due to the activation of fibroblasts by free radicals [75]. PgSE seems to overcome collagen accumulation directly or indirectly via the antioxidant constituents within it. For instance, a report indicated that pomegranate treatment inhibits collagen deposition in CCl_4_-induced liver toxicity [76]. This is particularly interesting because an increase in the thickness of the extracellular matrix has been correlated with male infertility [77].

Furthermore, the examination of the DNA ploidy indicated that the S phase population was diminished in the testes of tramadol-treated adult and adolescent rats. S phase is the phase of the cell cycle in which DNA is replicated and represents the proliferation index. This result means that tramadol might induce oxidative damage of proteins involved in cell cycle progression and ends up with p53 activation which in turn mediates apoptosis [21, 62, 78]. In contrast, the cotreatment of PgSE with tramadol showed a normal S phase distribution; this result could be explained by the evidence that pomegranate polyphenolics induce the cell cycle progression of the UV-induced S phase arrested fibroblast cells as previously identified [79].

Taken together, the presence of free radicals in testis is a normal physiological event; however, the increase in their synthesis stimulates the oxidative stress and DNA damage of cells. All of the above protective effects of pomegranate could be due to the prevention of free radicals generated by tramadol. We specifically tested an extract from pomegranate seeds which are commonly considered an agrowaste, while it has a valuable content of bioactive compounds. We measured the phenolic and flavonoids contents in addition to the free radical DPPH scavenging activity in the seed extract. Phenolic and flavonoid compounds are good electron donors because they hold an aromatic ring bearing at least one hydroxyl group which can directly terminate free radical chain reaction [80, 81]. Likewise, the DPPH free radical scavenging assay is a widely used assay to predict antioxidant activities based on the ability of certain substances to donate a hydrogen atom to the radical [82]. The measured values in PgSE are considered having a high antioxidant activity as compared with previous studies [83–89]. Consequently, this assumes that both total phenols and total flavonoids within PgSE possess a mutual activity to protect against tramadol-induced testicular toxicity. However, the PgSE had a complete protective effect on adults but partially on adolescent rats as revealed by TUNEL assay and DNA ploidy, reflecting that young age patients are more sensitive to analgesic drugs and that should be considered. It is recommended that patients on chronic tramadol therapy should be routinely screened for symptoms of gonadotoxicity.

In brief, our finding hypothesizes that PgSE supplementation may have a potential therapeutic role to overcome the toxic effects resulting from various analgesic drugs on long-term use. Further investigations with other analgesic drugs are necessary to confirm this hypothesis.

## 5. Conclusions

The chronic exposure to tramadol induces testicular damage in adult and adolescent rats. Histological and ultrastructural examinations revealed that tramadol induced hemorrhage of blood vessels, intercellular spaces, interstitial vacuoles, exfoliation of germ cells in lumen, cell apoptosis, chromatin degeneration of elongated spermatids, and malformation of sperm axonemes. Moreover, tramadol disrupted collagen metabolism and cell cycle progression. All of these adverse effects were protected by the PgSE cotreatment in adults and partially in adolescent rats. The phytochemistry of PgSE showed a high content of antioxidant compounds and high DPPH free radical scavenging activity.

## Data Availability

All data used to support the findings of this study are included within the article and the supplementary information files.

## Supplementary Materials

Supplementary Materials Figure S1: the experimental model used in the current study. Adult and adolescent male Wistar rats were examined as two different groups. Each group was divided into three subgroups as follows: control, Tr-treated, and Tr/PgSE-treated groups. The control group received orally 1 ml of 0.9% saline. The Tr-treated group received orally tramadol dose of 20, 40, and 80 mg/kg during the 1st, 2nd, and 3rd week, respectively. The Tr/PgSE-treated group received orally 40 mg/kg PgSE in addition to the tramadol dose. Tr: tramadol. PgSE: Pomegranate seed extract. Figure S2: histopathology of adult and adolescent rat testicular sections at low magnification. Sections of saline-treated controls, PgSE-treated controls, Tr-treated group, and Tr/PgSE-treated group were examined via HE staining. Scale bars: 100 *μ*M. Table S1: quantitative DNA ploidy of Tr-treated testicular tissues and cotreated with PgSE.
