# Correction to “Pomegranate Seeds Extract Possesses a Protective Effect against Tramadol‐Induced Testicular Toxicity in Experimental Rats”

**DOI:** 10.1155/bmri/9862436

**Published:** 2026-01-10

F. Minisy, H. Shawki, A. El Omri, A. Massoud, E. Omara, F. Metwally, M. Badawy, N. Hassan, N.S. Hassan, H. Oishi, “Pomegranate Seeds Extract Possesses a Protective Effect against Tramadol‐Induced Testicular Toxicity in Experimental Rats,” *BioMed Research International*, 2020, 2732958, https://doi.org/10.1155/2020/2732958.

In the article, there is an error in Figure 4, made during the preparation of the Figure and highlighted on PubPeer [1]. Specifically, the Adult and Adolescent control tissue panels contain repeated elements. The correct Figure 4 is shown below:

Figure 4Morphometrical analyses of Tr‐treated testicular tissues and cotreated with PgSE. (a) Adult and adolescent rat testes were examined via Masson′s trichrome staining. The blue color represents collagen density (star). The Tr‐treated group showed a significant increase in collagen fibers content in both adult and adolescent rats but not in the cotreated groups. Scale bars: 100 *μ*M. (b) The area percentage of collagen fibers measured in rat testes from each group was shown as bars. Data represent the mean ± SD,  ^∗∗^ *p* < 0.01. ns: nonsignificant.

**Figure:**
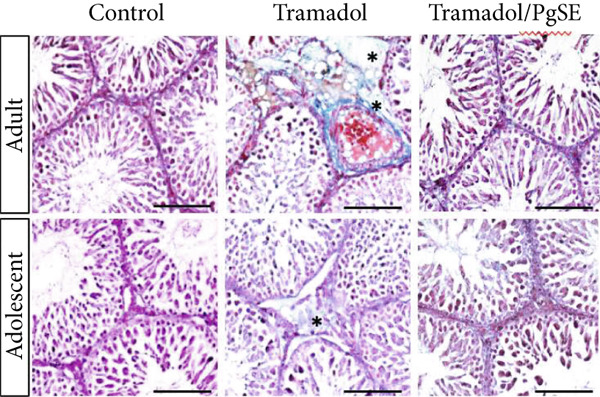
(a)

**Figure:**
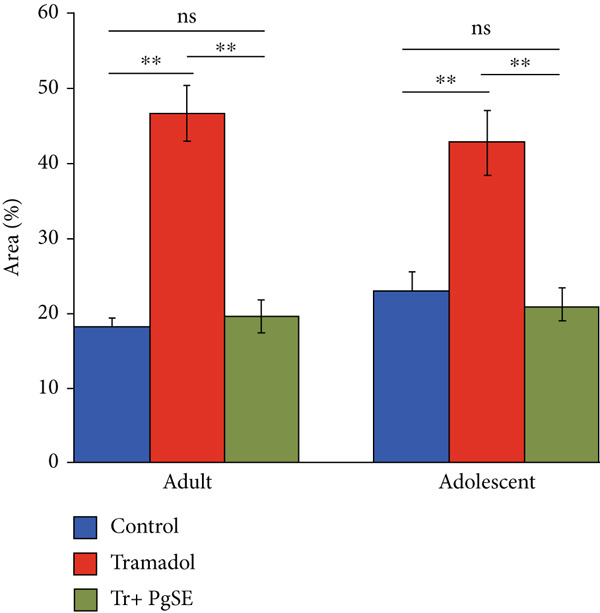
(b)

We apologize for this error.
