# Supplementary material for: Pomegranate Seeds Extract Possesses a Protective Effect against Tramadol-Induced Testicular Toxicity in Experimental Rats
Source: Biomed Res Int. 2020 Mar 9;2020:2732958. doi: 10.1155/2020/2732958 (PMC7085358; doi:10.1155/2020/2732958)
Supplement: Supplementary Materials — Figure S1: the experimental model used in the current study. Adult and adolescent male Wistar rats were examined as two different groups. Each group was divided into three subgroups as follows: control, Tr-treated, and Tr/PgSE-treated groups. The control group received orally 1 ml of 0.9% saline. The Tr-treated group received orally tramadol dose of 20, 40, and 80 mg/kg during the 1st, 2nd, and 3rd week, respectively. The Tr/PgSE-treated group received orally 40 mg/kg PgSE in addition to the tramadol dose. Tr: tramadol. PgSE: Pomegranate seed extract. Figure S2: histopathology of adult and adolescent rat testicular sections at low magnification. Sections of saline-treated controls, PgSE-treated controls, Tr-treated group, and Tr/PgSE-treated group were examined via HE staining. Scale bars: 100 μM. Table S1: quantitative DNA ploidy of Tr-treated testicular tissues and cotreated with PgSE. [file 2732958.f1.docx]

**Supporting Information**

# S1 Fig. The experimental model used in the current study. Adult and adolescent male Wistar rats were examined as two different groups. Each group was divided into three subgroups as following; Control, Tr-treated, and Tr/PgSE treated. The control group received orally 1 ml of 0.9% saline. The Tr-treated group received orally tramadol dose of 20, 40, and 80 mg/kg during the 1^st^, 2^nd^, and 3^rd^ week, respectively. The Tr/PgSE treated group received orally 40 mg/kg PgSE in addition to the tramadol dose. Tr; Tramadol. PgSE; Pomegranate seed extract.

**S2 Fig. Histopathology of adult and adolescent rat testicular sections at low magnification.** Sections of saline-treated controls, PgSE-treated controls, Tr-treated, and Tr/PgSE treated were examined via HE staining. Scale bars 100 µM.

**S1 Table.** Quantitative DNA-ploidy of Tr-treated testicular tissues and co-treated with PgSE.


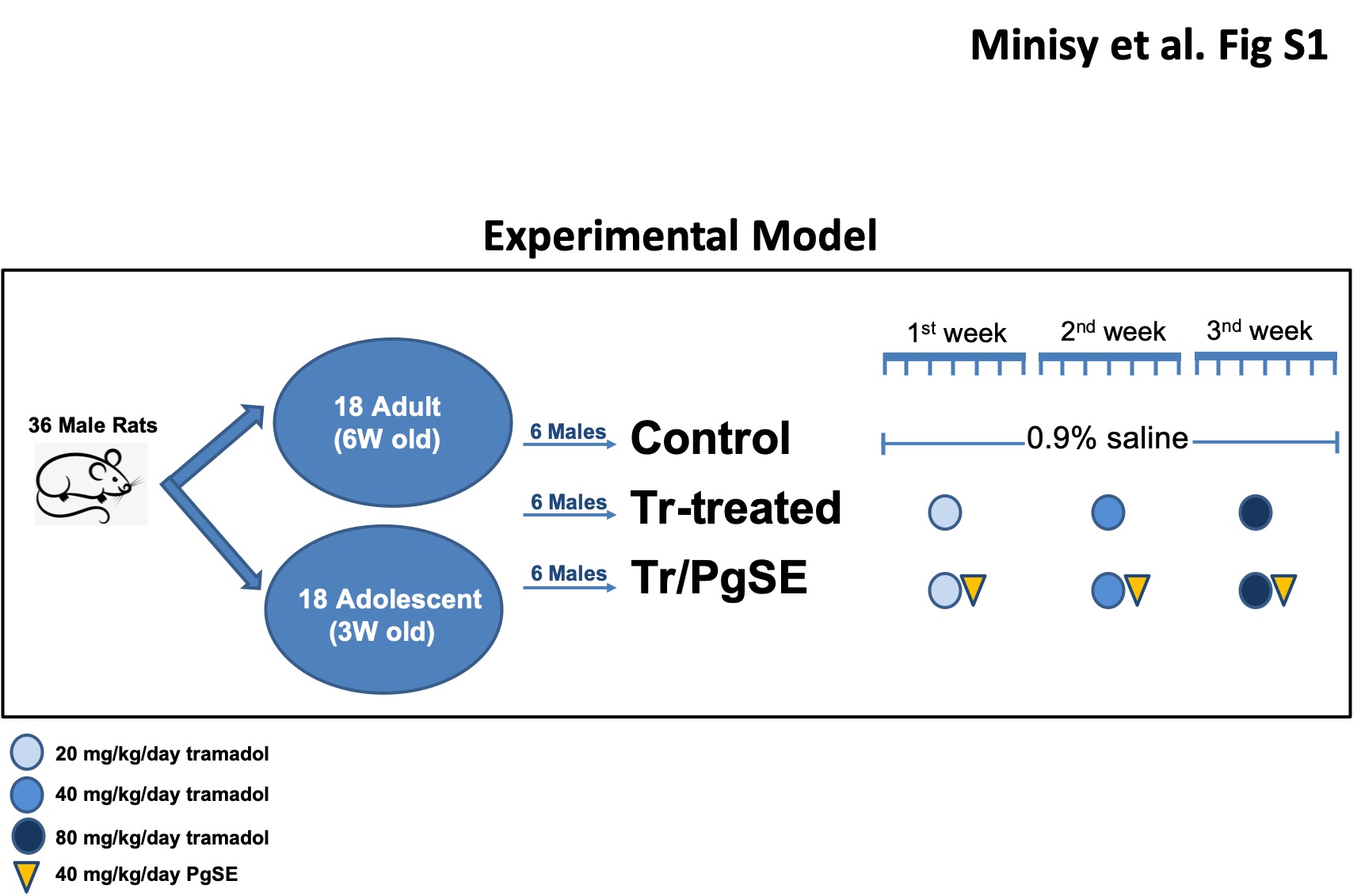


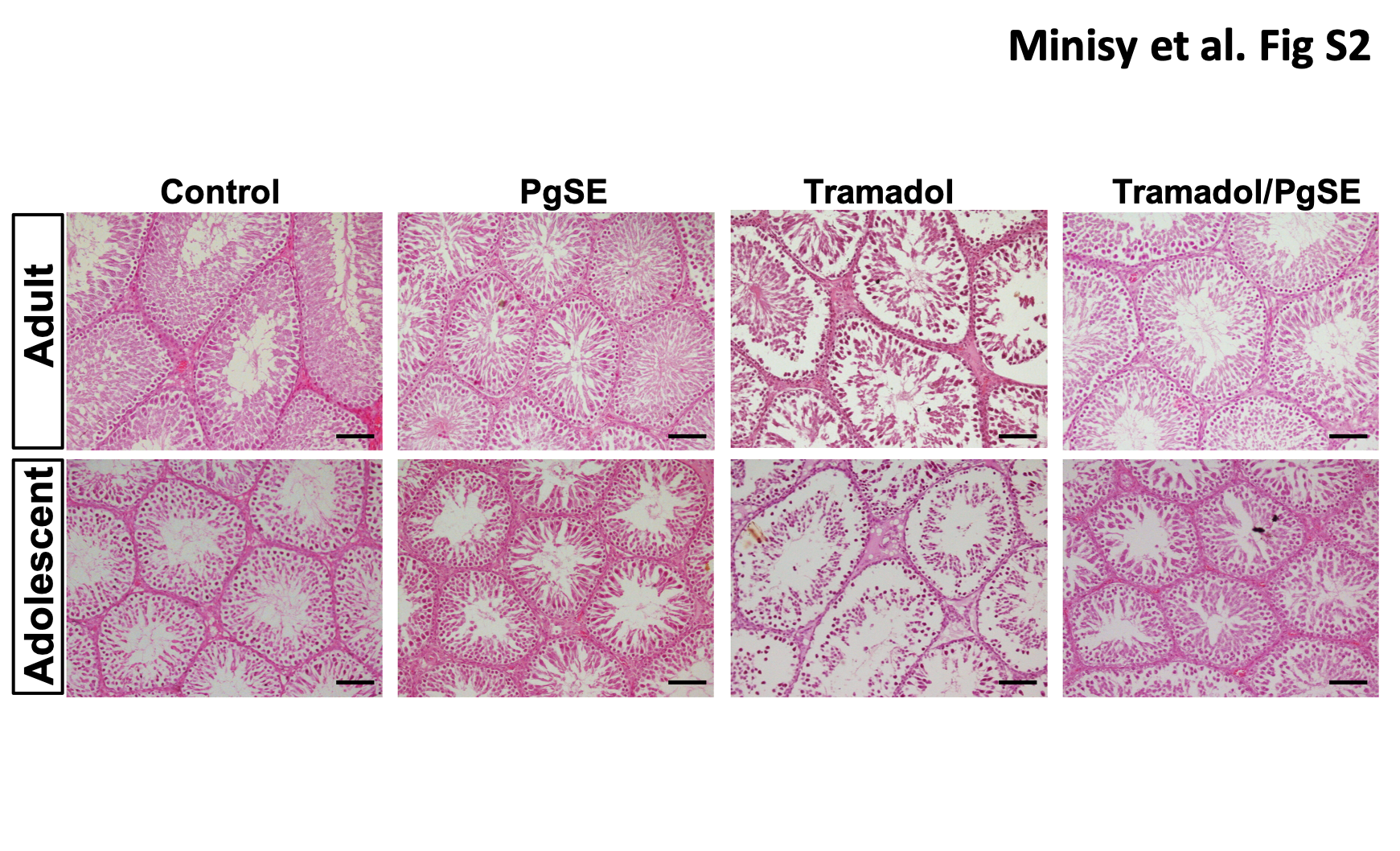


| **Range** | **Adult** | | | | | | **Adolescent** | | | | | | |
| --- | --- | --- | --- | --- | --- | --- | --- | --- | --- | --- | --- | --- | --- |
|  | **Control** | | **Tr** | | **Tr/PgSE** | | **Control** | | **Tr** | | **Tr/PgSE** | | |
|  | **% Cells** | **Mean±SD** | **% Cells** | **Mean±SD** | **% Cells** | **Mean±SD** | **% Cells** | **Mean±SD** | **% Cells** | **Mean±SD** | **% Cells** | **Mean±SD** |  |
| **Haploid**  **(< 1.5c)** | 9.74% | 1.27±0.15 | 60.00% | 1.12±0.21 | 40.71l% | 1.24±0.16 | 9.65% | 1.36±0.12 | 52.94% | 1.13±0.22 | 47.58% | 1.17±0.24 |  |
| **Diploid**  **(1.5c-2.5c)** | 74.34% | 1.98±0.26 | 31.30% | 1.93±0.30 | 42.48% | 1.92±0.29 | 69.29% | 1.94±0.26 | 42.86% | 1.90±0.26 | 45.97% | 1.94±0.29 |  |
| **Triploid**  **(2.5c-3.5c)** | 15.04% | 2.88±0.22 | 6.96% | 2.96±0.36 | 15.04% | 3.01±0.32 | 20.18% | 2.81±0.20 | 3.36% | 2.95±0.26 | 6.45% | 2.83±0.28 |  |
| **Tetraploid**  **(3.5c-4.5c)** | 0.89% | 3.75±0.0 | 1.74% | 3.70±0.10 | 1.77% | 3.91±0.47 | 0.88% | 3.98±0.00 | 0.84% | 3.61±0.00 | 0.00% | -- |  |

**S1 Table**
